# Something in Mechanical Dentistry

**Published:** 1876-08

**Authors:** J. Hardman

**Affiliations:** Iowa


### SOMETHING IN MECHANICAL DENTISTRY.

BY J. HARDMAN, D. D. S., IOWA.

It is true that celluloid is very much taking the place of vulcanized rubber for the basis of artificial dentures. This, no doubt, is largely owing to the unreasonable treatment dentists have received from the Vulcanite Company, and not because celluloid is superior. It is less strong and dense, will wear away faster and lose its polish earlier. But it is lighter, more easily moulded and finished up. The dry heat plan is, no doubt, the best for any variety of work it may be used for. And I would say to those who have the old style oil apparatus, that they can do just as good work with it as with any of those intended for dry heat alone. Get it clean and put no fluids of any kind into the chamber. If the thermometor will not permit running the heat up to 325° or 330° remove it when nearly up and proceed carefully.

I am well convinced that the profession encourages entirely too much the use of these cumbersome plastic materials, and I would insist that each dentist will resolve to acquaint himself with the use of gold, platina and silver, especially for partial sets. When patients are unable to pay for gold or platina, silver is really far superior to either rubber or celluloid, especially so where the gums are full and the teeth short.

We meet cases that are well adapted to platina, put up in the same manner as gold plate, taking care to stiffen by soldering duplicate strips where needed, and there is probably nothing so good for backings, either for gold or platina plate, as platina. Its pliability renders it quite suitable, and readily fitted to the artificial tooth. And then a very much finer and higher flowing solder can be used, as there need be no fears of melting the backing by over heat.

Continuous gum work is only suitable, or financially feasible, for dentists practicing where people can afford to pay high prices. The repair of this varity of work is difficult and generally quite expensive. Its greater weight makes it more suitable for lower than upper sets. It is, however, quite pure and cleanly in the mouth.

Pure tin, nine parts hardened by one part bismuth will make a very good lower base, where economy must be practiced.

